# *Helicobacter pylori* Infection in Gastroesophageal Reflux Disease in the Asian Countries

**DOI:** 10.1155/2015/985249

**Published:** 2015-01-06

**Authors:** Su Jin Hong, Sang Woo Kim

**Affiliations:** ^1^Digestive Disease Center and Research Institute, Department of Internal Medicine, Soonchunhyang University College of Medicine, Bucheon, Republic of Korea; ^2^Department of Internal Medicine, Gastrointestinal Center, Medical College, Catholic University of Korea, 222 Banpo-daero, Seocho-gu, Seoul 137-701, Republic of Korea

## Abstract

*Helicobacter pylori* infection, a common infection in many countries, is related to the clinical course of upper gastrointestinal diseases. Gastroesophageal reflux disease (GERD) is a common esophageal disease in Western countries and its prevalence is increasing in Asian countries. The pathophysiology of GERD is multifactorial. Although no single factor has been isolated as the cause of GERD, a negative association between the prevalence of *H. pylori* and the severity of GERD, including Barrett's esophagus, has been demonstrated in epidemiological studies. The high prevalence of *H. pylori* infection affects the incidence of GERD in Asian countries. In the subjects with East Asian CagA-positive strains, acid injury may be minimized by hypochlorhydria from pangastritis and gastric atrophy. Additionally, host genetic factors may affect the development of GERD. The interactions between genetic factors and the virulence of *H. pylori* infection may be the reason for the low prevalence of GERD in Asian countries. *H. pylori* eradication is not considered pivotal in GERD exacerbation based on evidence from Western studies. A recent meta-analysis demonstrated that eradication therapy of *H. pylori* was related to a higher risk of developing *de novo* GERD in Asian studies. *H. pylori* infection remains an inconclusive and important issue in GERD in Asian countries.

## 1. Introduction

The prevalence of gastroesophageal reflux disease (GERD) in the general population has been estimated to be 10–20% [1–4]. Conversely, most Asian population-based studies have reported a lower prevalence of less than 10% [3–6]. In epidemiological studies,* H. pylori* and GERD have been found to be negatively associated and strongly related to cytotoxin-associated gene product- (CagA-) positive strains of* H. pylori* [7]. However, an increasing prevalence of GERD and decreasing prevalence of* H. pylori* have been reported in Asian countries [8], which is in agreement with a previous report of no increase in the prevalence of GERD symptoms with age [4]. GERD markedly reduces patients' quality of life and imparts a significant economic burden on the healthcare system [9–11]. Therefore, decreasing the prevalence of* H. pylori* infection is an important issue in GERD, especially in Asian populations. In addition,* H. pylori* eradication has been presumed to exacerbate GERD due to improvement of gastritis and the recovery of hypochlorhydria; several studies have been conducted to clarify this controversy.

## 2. Gastric Acidity and* H. pylori*

Gastric acid plays a key role in the etiology of GERD and is an element of the disease that may be modified by* H. pylori* infection. Gastric secretion can increase, decrease, or remain steady depending on the pattern of* H. pylori*-related inflammation [12]. The major components of acid secretion in patients with* H. pylori* infection include the density of* H. pylori* colonization, its distribution, and the severity of the mucosal inflammatory response to the infection. Patients with a duodenal ulcer and* H. pylori* infection have antrum-predominant gastritis, which leads to hypergastrinemia and acid hypersecretion. In contrast, patients with gastric ulcer or gastric cancer present mainly with corpus-predominant gastritis or pangastritis, which is characterized by intense destruction or atrophy of acid-secreting glands. Patients who have corpus-predominant gastritis or pangastritis also show gastric acid hyposecretion [13, 14]. Bacterial virulence and host inflammatory responses are important in determining patterns of acid secretion and gastritis. East Asian CagA-positive strain of* H. pylori* induces primarily corpus-predominant gastritis or pangastritis with hypochlorhydria. And, East Asian CagA-positive strain is strongly associated with gastric cancer. A Japanese study revealed different sequences of CagA between the regions where gastric cancer is prevalent or not. The authors defined the East Asian CagA-positive strains which showed the specific repeat sequences located in the 3′ region of* cagA* gene. In the study, most CagA-positive strains in Asian countries were East Asian CagA-positive strains and most CagA-positive strains in Western countries were Western CagA-positive strains [15]. In Asian populations with East Asian CagA-positive strains, acid injury may be minimized by hypochlorhydria from pangastritis and gastric atrophy. Additionally, host genetic factors may affect the development of GERD. IL-1B and IL-1RN genetic polymorphisms are inversely associated with the risk of GERD in* H. pylori*-infected subjects because their specific genotypes are linked to corpus atrophy, gastric cancer, and hypochlorhydria [16–20]. Thus, such specific genotypes, including the IL-1B-511-T, IL-1B-31-C, and IL-1RN-1 alleles, can be considered protective against GERD. In particular, the subjects with IL-1B-511 T allele is accentuated in the presence of* H. pylori* infection due to high gastric mucosal IL-1*β* levels [20, 21]. However, other investigators have reported contradictory results that IL-1B-511-T allele was associated with reflux esophagitis [22]. These opposite results suggest ethnic differences regarding IL-1 genetic polymorphisms and levels of gastric mucosal IL-1*β*. Additionally, several genetic risk factors for GERD, including polymorphisms in G-protein beta 3 subunit gene (GNB3) [23], IL-10 [24], CYP2C19 [25], glutathione S-transferase P1 [26, 27], cyclin D1 [28], and DNA repair genes [29], may be involved.

The interactions between genetic factors and the virulence of* H. pylori* infection may be the reason for the low prevalence of GERD in Asian countries [16–18, 20–22, 27, 29].

## 3. Epidemiological Evidence of a Link between* H. pylori* Infection and GERD

Table 1 shows recent epidemiological reports of an inverse relationship between* H. pylori* infection and reflux esophagitis or Barrett's esophagus in the western countries and East Asian countries [30–35]. This negative association was also evident in patients with severe GERD and* H. pylori* infection with virulent CagA-positive strains in Western countries [36, 37]. The prevalence of* H. pylori* infection is inversely correlated with the risk and severity of reflux esophagitis; [30, 37, 38] and the prevalence of* H. pylori* infection suggests a protective role in both Barrett's esophagus and esophageal adenocarcinoma [7, 34, 35, 37–41].

## 4. Proton Pump Inhibitors (PPIs) in GERD Patients with* H. pylori* Infection

Long-term maintenance therapy of proton pump inhibitors (PPIs) for GERD induces gastritis and progression of gastric atrophy and intestinal metaplasia to gastric adenocarcinoma in patients with* H. pylori* infection [46, 47]. These patterns are significantly associated with the CagA-positive strains [48]. Current guidelines, including the Asia-Pacific Consensus for* H. pylori* infection, recommend* H. pylori* eradication in GERD patients requiring long-term PPIs [49]. However, there is no evidence that* H. pylori* eradication reduces the risk of gastric adenocarcinoma in patients with this condition.

## 5. *H. pylori* Eradication in GERD

Despite the inverse relationship between* H. pylori* and GERD in cross-sectional studies, the results are less consistent in prospective studies of* H. pylori* eradication in patients with GERD. Early studies revealed that* H. pylori* eradication was positively associated with reflux esophagitis or GERD symptoms in patients with gastric and duodenal ulcer diseases [50, 51]. Hiatal hernia, corpus gastritis, and CagA-positive* H. pylori* strains have been reported to be risk factors for newly developed reflux esophagitis after* H. pylori* eradication [51, 52]. However, other studies have shown improvement of reflux symptoms after* H. pylori* eradication in patients with peptic ulcer disease and nonulcer dyspepsia [53, 54]. The Maastricht IV Consensus Report suggested that* H. pylori* eradication does not exacerbate preexisting GERD or affect treatment efficacy [55]. A recent meta-analysis demonstrated that eradication therapy of* H. pylori* was related to a significantly higher risk of developing* de novo* GERD in Asian studies [42]. In contrast, no such risk has been reported by Western studies [43–45].  Table 2 shows the summaries of the results of meta-analyses. However, this remains an inconclusive issue in Asian countries. For example, two large-scale cohort studies in Korea produced inconsistent results [56, 57]. Thus, the revised version of the Korean guidelines for* Helicobacter pylori* infection states that* H. pylori* eradication does not affect the development or clinical course of GERD [58].

## 6. Conclusion

*H. pylori* infection and GERD are highly prevalent conditions globally. The prevalence of* H. pylori* varies geographically and among ethnicities. Many epidemiological studies have shown a negative correlation between* H. pylori* infection and GERD. A specific virulence factor, such as CagA, and specific host genotypes may affect the diverse prevalence and other aspects of GERD owing to individual differences in acid secretion. A high prevalence of CagA-positive strains has been reported in Asian countries. The diversity of* H. pylori* infection between Western and Asian countries should be considered when analyzing the results of studies of* H. pylori* eradication in GERD patients. To date, cohort studies and randomized controlled trials of the effects of* H. pylori* eradication on GERD are inconclusive. The decreasing prevalence of* H. pylori* and the recovery of acid secretion capacity after eradication in patients with CagA-positive* H. pylori* and corpus gastritis are possible causes of the higher prevalence of GERD in Asian countries. These issues necessitate a more detailed study.

## Figures and Tables

**Table 1:** Recent epidemiologic studies for association between *H*. *pylori* infection and GERD.

Study [references]	Type of study	Location	Number of cases in each group (*n*)	*H*. *pylori* infection assessments	*H*. *pylori* prevalence (%) in each group
Chung et al. 2011 [30]	Case-control	Korea	Reflux esophagitis (2,808) Control (2,808)	Serology	Reflux esophagitis (38.4) Control (58.2)

Gunji et al. 2011 [31]	Cross-sectional	Japan	Erosive esophagitis (1,831) No erosive esophagitis (8,009)	Serology	Erosive esophagitis (13.6) No erosive esophagitis (33.4)

Chiba et al. 2012 [32]	Cross-sectional	Japan	Erosive esophagitis (728) No erosive esophagitis (4,262)	Serology	Erosive esophagitis (9.4) No erosive esophagitis (14.9)

Ashktorab et al. 2012 [33]	Case-control	USA	Reflux esophagitis (58) Gastritis (1,558) Reflux esophagitis and gastritis (363) Normal control (41)	Biopsy silver stain or immunohistochemistry	Reflux esophagitis (3.8) Gastritis (40) Reflux esophagitis and gastritis (34) Normal control (34)

Sonnenberg et al. 2010 [34]	Cross-sectional	USA	Barrett's esophagus (2,510) No Barrett's esophagus (76,475)	Biopsy immunohistochemistry	Barrett's esophagus (5.7) No Barrett's esophagus (12.2)

Thrift et al. 2012 [35]	Case-control	Australia	Simple Barrett's esophagus (217) Dysplastic Barrett's esophagus (95) Control (398)	Serology	Simple Barrett's esophagus (12) Dysplastic Barrett's esophagus (3) Control (18)

GERD: gastroesophageal reflux disease.

**Table 2:** Results of meta-analyses for *Helicobacter pylori* eradication on GERD.

Study [references]	Number of enrolled studies	Location of enrolled studies	Risk ratio (95% confidence interval)	Conclusion
Xie et al. 2013 [42]	12 cohort studies and 12 RCTs	*Cohort* Europe: 4 North America: 1 Asia: 7 *RCTs* Europe: 7 South America: 1 Asia: 4	3 type A cohort studies: 2.50 (1.46–4.26, *P* = 0.0008) 9 type B cohort studies: 1.70 (1.30–2.23, *P* = 0.0001) 12 RCTs: 1.09 (1.23–3.22, *P* = 0.005) 4 Asian RCTs: 4.53 (1.66–12.36, *P* = 0.003)	Eradication of the infection may be a risk factor for *de novo* endoscopic GERD, especially in Asian populations.

Yaghoobi et al. 2010 [43]	5 cohort studies and 7 RCTs	*Cohort* Europe: 1 Asia: 4 *RCTs* Europe: 3 North America: 3 South America: 1	5 cohort studies: 1.37 (0.89–2.12, *P* = 0.15) 6 RCTs using erosive GERD as outcome: 1.11 (0.81–1.53, *P* = 0.52) 5 RCTs using symptomatic GERD as outcome: 1.22 (0.89–1.69, *P* = 0.22)	There is no association between *H*. *pylori* eradication and development of new cases of GERD in the population of dyspeptic patients.

Qian et al. 2011 [44]	11 RCTs	Europe: 5 North America: 3 South America: 1 Asia: 1 Multinational: 1	7 RCTs using heartburn symptom as outcome: 0.88 (0.63–1.23, *P* = 0.46) 10 RCTs using erosive esophagitis as outcome: 0.97 (0.67–1.40, *P* = 0.88)	*H*. *pylori* eradication does not aggravate the clinical outcomes in terms of short-term and long-term posteradication occurrence of GERD.

Saad et al. 2012 [45]	10 RCTs	Europe: 7 North America: 2 Asia: 1	10 RCTs using symptomatic GERD as outcome: 0.81 (0.56–1.71, *P* = 0.27) 10 RCTs using endoscopic esophagitis as outcome: 1.13 (0.72–1.78, *P* = 0.59)	Treatment of *H*. *pylori* does not seem to increase GERD symptoms or reflux esophagitis. However, documented eradication of *H*. *pylori* appears to significantly improve GERD symptoms.

RCT: randomized controlled trial; GERD: gastroesophageal reflux disease; *H*. *pylori*: *Helicobacter pylori*.
